# Clinical stratification utility of HER2, PD-L1, MSI/MMR, and CLDN18.2 in conversion therapy for gastric cancer: narrative review

**DOI:** 10.3389/fonc.2026.1888941

**Published:** 2026-08-26

**Authors:** Tao Ding, Peiqiang Chen, Huajiong Yu

**Affiliations:** 1 Graduate School, Zhejiang Chinese Medical University, Hangzhou, China; 2 Shulan (Hangzhou) Hospital Affiliated to Zhejiang Shuren University Shulan International Medical College, Hangzhou, China

**Keywords:** advanced gastric cancer, Claudin18.2, conversion therapy, HER2, immunotherapy, MSI/MMR, PD-L1, targeted therapy

## Abstract

Most patients with gastric cancer considered for conversion therapy present with locally advanced or metastatic disease, where surgery alone or conventional chemotherapy often provides limited benefit. Conversion therapy aims to reduce tumor burden through systemic treatment, reassess treatment response over time, and create an opportunity for radical surgery with complete tumor removal. This approach is mainly considered for patients with initially unresectable, borderline resectable, or limited metastatic disease. However, because not all patients benefit from this strategy, careful selection of those most likely to respond to systemic therapy and proceed to surgery is essential. With the development of immunotherapy and targeted therapy, conversion therapy for gastric cancer has shifted from empiric chemotherapy toward biomarker-guided individualized treatment. HER2 status may guide anti-HER2 therapy, PD-L1 may help identify patients who benefit from immunotherapy combined with chemotherapy, MSI-H/dMMR suggests greater sensitivity to immunotherapy, and CLDN18.2 provides a new targeted option, particularly for HER2-negative disease. This review examines the clinical value of HER2, PD-L1, MSI/MMR, and CLDN18.2 in treatment selection, response prediction, repeated reassessment, and surgical timing. These biomarkers may help identify patients with treatment-sensitive disease who warrant surgical reassessment. However, because most supporting evidence is extrapolated from palliative/metastatic or perioperative trials, such evidence should not be interpreted as demonstrating improved conversion-surgery or R0-resection rates, pathological response, or postoperative disease-free survival in conversion-therapy populations; surgical eligibility remains dependent on comprehensive clinical and multidisciplinary assessment.

## 1 Introduction

Gastric cancer remains a major gastrointestinal malignancy worldwide and continues to impose a substantial disease burden in several Southeast Asian countries. Many patients are diagnosed at a locally advanced or metastatic stage, where tumor heterogeneity, peritoneal spread, extensive lymph node involvement, and treatment resistance limit the benefit of surgery alone or conventional chemotherapy (1). Although palliative systemic therapy remains the standard approach for many patients with advanced disease, a selected subgroup may become eligible for surgery after an effective treatment response.

With the development of multidisciplinary care, conversion therapy has emerged as a potential strategy for selected patients with initially unresectable, marginally resectable, or oligometastatic gastric cancer. According to the KINGCA WEEK 2024 expert consensus, conversion therapy is defined as a shift from palliative systemic treatment to curative-intent surgery of the primary and/or metastatic tumors, aiming for R0 resection of disease initially considered technically or oncologically unresectable or marginally resectable (2). Unlike neoadjuvant therapy for clearly resectable tumors, its clinical value depends not only on tumor shrinkage but also on careful reassessment, appropriate surgical timing, and the feasibility of complete resection.

Recent advances in immunotherapy and targeted therapy have further expanded treatment options for advanced gastric cancer. These developments have shifted conversion therapy from a mainly chemotherapy-based approach toward more individualized treatment guided by molecular biomarkers. Among these biomarkers, HER2, PD-L1, MSI/MMR, and CLDN18.2 have particular clinical relevance because they may help guide treatment selection, predict response, and identify patients who are more likely to benefit from conversion therapy. This review summarizes recent progress in conversion therapy for locally advanced and advanced gastric cancer, with a focus on the clinical stratification value of these key biomarkers.

## 2 Methods

The literature search was conducted on 10 January 2026 and updated on 6 August 2026. PubMed, Web of Science, and Google Scholar were searched from inception to 6 August 2026. Disease terms included “gastric cancer”, “gastric adenocarcinoma”, and “gastroesophageal junction cancer”; treatment terms included “conversion therapy”, “conversion surgery”, and “downstaging”. Biomarker terms included HER2/ERBB2, PD-L1/CPS/TAP, MSI/MMR, and CLDN18.2/claudin 18.2. Reference lists were manually screened, and regulatory and guideline sources were consulted to verify current indications and biomarker thresholds. The search yielded 313 records. After removing 78 duplicates, 235 titles and abstracts were screened, and 82 publications underwent full-text assessment. English-language clinical trials, observational studies, reviews, guidelines, consensus statements, and conference abstracts with otherwise unavailable clinically relevant data were eligible. Sources had to address conversion therapy or surgery, biomarker-guided systemic treatment, response, resectability, or outcomes in locally advanced, initially unresectable, or metastatic gastric/GEJ adenocarcinoma. Duplicate reports, non-English publications, preclinical studies, case reports, and sources lacking clinically relevant treatment or outcome data were excluded. After full-text assessment, 31 publications were excluded, leaving 51 cited sources. Tao Ding and Peiqiang Chen independently screened titles and abstracts and assessed full texts. Disagreements were resolved through discussion or, when needed, by Huajiong Yu. Evidence was synthesized qualitatively, prioritizing prospective trials, guidelines or consensus statements, and large multicenter studies. Conversion-specific studies were evaluated by conversion-surgery rate, R0 resection, pathological response, and postoperative survival. Palliative/metastatic and perioperative studies were treated as indirect evidence of treatment activity or biomarker-defined sensitivity. Their ORR, PFS, OS, and neoadjuvant pCR results were not interpreted as direct evidence of conversion benefit.

## 3 Results

### 3.1 Definition, patient selection, and therapeutic goals of conversion therapy for advanced gastric cancer

Conversion therapy is an integrated, multidisciplinary strategy designed for patients with locally advanced or advanced gastric cancer who are not amenable to upfront curative resection or are expected to have a low likelihood of complete tumor clearance (3). Unlike palliative therapy, its purpose is not limited to disease control; rather, it aims to reduce tumor burden, control metastatic lesions, and create an opportunity for R0 resection after effective systemic treatment.

Potential candidates include patients with unresectable or borderline resectable locally advanced disease and selected patients with limited metastatic disease, such as limited liver metastasis, distant lymph node metastasis, positive peritoneal cytology, or low-volume peritoneal metastasis (4). Surgery may be reconsidered only when both the primary tumor and metastatic lesions are well controlled and when all visible disease can be completely removed or effectively managed. Patients with extensive peritoneal metastasis, obvious ascites, rapid disease progression, poor general condition, or diffuse-type/signet-ring cell carcinoma require more cautious evaluation (5).

Chemotherapy remains the foundation of conversion therapy. Fluoropyrimidine- and platinum-based regimens, including SOX, XELOX, and FOLFOX, are commonly used. Recent progress in immunotherapy and molecularly targeted agents has increased the available therapeutic options and accelerated the transition of gastric cancer conversion therapy toward biomarker-based decision-making. PD-L1 and MSI/MMR status may help guide the use of immunotherapy combined with chemotherapy, while HER2 and CLDN18.2 can identify patients who may benefit from targeted combination strategies (6, 7).

The decision to proceed to surgery should be based on repeated clinical assessment and multidisciplinary discussion, rather than tumor shrinkage on imaging alone. Assessment should include regression of the primary tumor, control of metastatic disease, peritoneal status, lavage cytology, tumor markers, nutritional status, and surgical fitness. Conversion surgery is most likely to provide benefit when systemic disease is controlled, no new uncontrolled lesions are present, and complete resection is considered feasible (8, 9).

Taken together, recent progress in gastric cancer conversion therapy is best understood as a shift in both systemic treatment and clinical decision-making rather than as the introduction of a single regimen. Early evidence from AIO-FLOT3 and subsequent multicenter cohorts, including CONVO-GC-1, demonstrated the feasibility of induction systemic therapy followed by curative-intent surgery in carefully selected patients with limited metastatic burden (4, 8). More recently, the systemic treatment backbone has expanded from fluoropyrimidine-platinum chemotherapy to immunotherapy-containing and biomarker-matched combinations directed against HER2 or CLDN18.2. Although these trials were not specifically designed to evaluate conversion therapy, their improvements in systemic disease control provide a rationale for surgical reassessment in selected responders (6, 7, 9). In parallel, the field has moved from relying primarily on radiological tumor shrinkage to a dynamic multidisciplinary process that repeatedly evaluates the primary and metastatic lesions, peritoneal status, emergence of new disease, patient fitness, and technical feasibility of R0 resection (2, 5, 8). Nevertheless, most evidence for newer systemic regimens is derived from unresectable or metastatic disease rather than conversion-specific randomized trials; these treatments should therefore be regarded as increasing conversion potential, whereas the survival benefit attributable to subsequent surgery still requires prospective confirmation.

**Figure 1:**
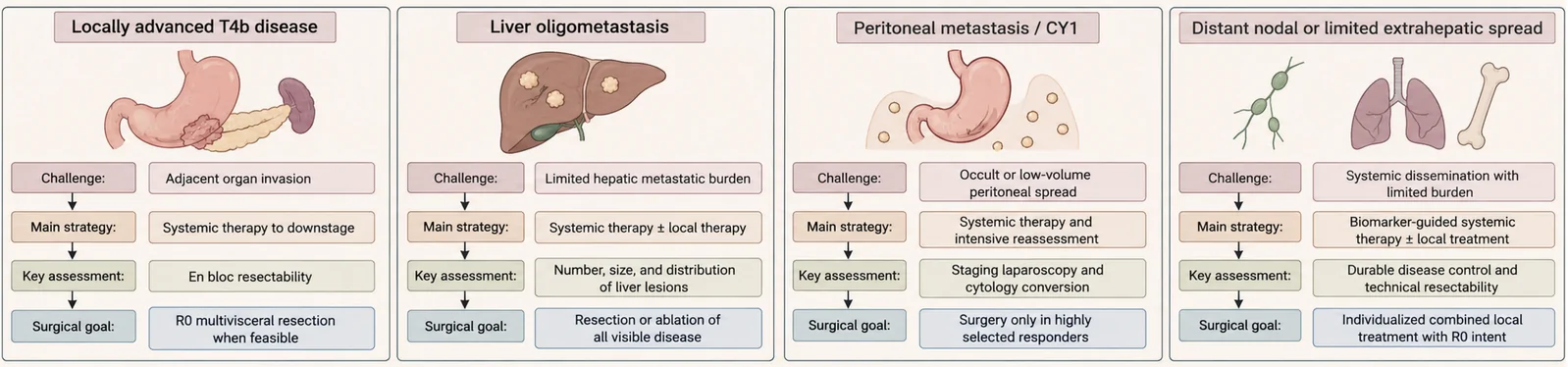
Metastasis pattern–based conversion therapy strategies in advanced gastric cancer. Four clinical scenarios are presented: locally advanced T4b disease, liver oligometastasis, peritoneal metastasis/CY1, and distant nodal or limited extrahepatic spread. For each scenario, the figure summarizes the main challenge, treatment strategy, reassessment focus, and surgical goal. CY1, positive peritoneal cytology; R0, microscopically margin-negative resection.

Nevertheless, conversion-specific prospective evidence is emerging. In a single-arm phase II study of 47 patients with stage IV gastric cancer, sintilimab combined with nab-paclitaxel/S-1 and apatinib achieved an R0 conversion rate of 51.1%, with median event-free and overall survival of 15.3 and 25.7 months, respectively (10). Preliminary results from the prospective FDZL-001 phase II trial further reported an R0 resection rate of 75.0% after camrelizumab plus Nab-POF in initially unresectable locally advanced or limited metastatic gastric/gastroesophageal junction adenocarcinoma (11). Consistent with the KINGCA WEEK 2024 consensus (2), these findings support individualized regimen selection and surgical reassessment at the point of maximal response. However, the small, single-arm designs and heterogeneous selection criteria preclude establishment of a standard regimen or validation of individual biomarkers as predictors of R0 conversion (**Figure 1**).

### 3.2 Clinical stratification value of four major molecular biomarkers in conversion therapy

#### 3.2.1 Stratification value of HER2 in conversion therapy for gastric cancer

HER2, encoded by the ERBB2 gene, is one of the earliest predictive biomarkers used in gastric cancer. HER2 is a transmembrane receptor tyrosine kinase that promotes tumor-cell proliferation through downstream PI3K-AKT and MAPK signaling following ERBB2 amplification or protein overexpression (12, 13). Unlike breast cancer, HER2 expression in gastric cancer is often heterogeneous, with incomplete membranous staining and possible discordance between primary and metastatic lesions. Because HER2 expression may change under treatment pressure, repeat testing of an accessible metastatic or progressing lesion should be considered when feasible, particularly before subsequent HER2-directed therapy (14). Therefore, standardized testing is essential, and HER2 positivity is generally defined as IHC 3+ or IHC 2+ with ISH/FISH positivity (12). HER2 positivity is more frequently observed in intestinal-type, well-differentiated, and gastroesophageal junction adenocarcinomas, making it an important marker for identifying patients who may benefit from anti-HER2-based systemic therapy.

Based on evidence from advanced gastric cancer rather than conversion-specific randomized trials, we interpret HER2 positivity as a factor that may increase the probability of deep tumor regression and subsequent surgical reassessment. In the ToGA trial, trastuzumab plus chemotherapy prolonged median overall survival from 11.1 to 13.8 months compared with chemotherapy alone (HR 0.74) (7). For initially unresectable, borderline resectable, or oligometastatic HER2-positive disease, this improvement in systemic disease control may create an opportunity for R0 resection, although HER2 positivity itself should not be regarded as an independently validated indication for conversion surgery.

Recent studies have further expanded HER2-directed strategies, including the design and earlier analyses of KEYNOTE-811 (15–17). Trastuzumab remains the backbone of anti-HER2 therapy. At the final analysis of KEYNOTE-811, pembrolizumab combined with trastuzumab and chemotherapy significantly improved overall survival in HER2-positive advanced gastric or gastroesophageal junction adenocarcinoma (HR 0.80; 95% CI, 0.67–0.94; P = 0.004) (18). However, no OS improvement was observed in the exploratory PD-L1 CPS <1 subgroup, which accounted for approximately 15% of patients (HR 1.10; 95% CI, 0.72–1.68); accordingly, the March 2025 FDA indication was restricted to patients with PD-L1 CPS ≥1 (18, 19). Trastuzumab deruxtecan has also shown efficacy in previously treated HER2-positive advanced gastric cancer, providing a potential option for later-line or reconversion therapy (20). However, HER2 status alone cannot determine surgical indications. Conversion surgery should be considered only when systemic disease is controlled, no new metastases appear, and R0 resection is judged feasible by multidisciplinary assessment.

#### 3.2.2 Stratification value of PD-L1 expression and its scoring systems in conversion therapy for gastric cancer

PD-L1 expression, rather than the combined positive score (CPS) or tumor area positivity (TAP) score itself, constitutes the predictive biomarker. Biologically, the binding of PD-L1 to PD-1 suppresses T-cell activation and facilitates tumor immune escape. CPS is one of several immunohistochemistry-based scoring algorithms used to quantify PD-L1 expression. It is calculated as the number of PD-L1-positive tumor cells, lymphocytes, and macrophages divided by the total number of viable tumor cells and multiplied by 100 (21). Thus, CPS reflects PD-L1 expression in both tumor and tumor-associated immune cells rather than representing an independent biomarker. TAP is another immunohistochemistry-based scoring algorithm. It estimates the percentage of the total tumor area, including tumor tissue and desmoplastic stroma, occupied by PD-L1-stained tumor cells and tumor-associated immune cells. Unlike the cell-count-based CPS algorithm, TAP is an area-based measurement. Therefore, CPS and TAP are distinct scoring algorithms, and their respective thresholds should not be considered directly interchangeable. Throughout this review, PD-L1 expression is considered the biomarker, whereas CPS and TAP refer to the scoring algorithms used in the respective clinical trials. The antibody assay, scoring algorithm, specimen site and timing, and trial- or treatment-specific cutoff should therefore be explicitly reported. CPS also has important analytical limitations. The 22C3 and 28–8 assays are not uniformly interchangeable at clinically relevant cutoffs, and spatial heterogeneity may lead to specimen-dependent classification (22). Thresholds are also treatment- and region-specific: the current EMA indication for first-line nivolumab plus chemotherapy requires CPS ≥5, whereas the current US FDA indication requires CPS ≥1 (23, 24). Thus, no single cutoff is universally applicable, and the antibody clone, specimen site and timing, scoring method, and cutoff should be reported when interpreting CPS.

Based on evidence from advanced-disease trials rather than conversion-specific studies, we interpret higher PD-L1 expression, as quantified using the trial-specific scoring algorithms employed in the cited studies, as a factor associated with a greater likelihood of deep and durable response to chemoimmunotherapy. Evidence from the phase III CheckMate 649 trial and its 5-year follow-up supports the use of nivolumab plus chemotherapy in advanced disease (25, 26). In the PD-L1 CPS ≥5 population, the 5-year overall survival rate was 16% with nivolumab plus chemotherapy versus 6% with chemotherapy alone, and the ORR was 60% versus 45%; in the intention-to-treat population, the ORR was 58% with nivolumab plus chemotherapy versus 46% with chemotherapy alone (25). KEYNOTE-859 also demonstrated improvements in overall survival, progression-free survival, and objective response rate, with greater benefit observed in patients with higher CPS values (27). The phase III RATIONALE-305 trial enrolled patients irrespective of PD-L1 expression. PD-L1 expression was prospectively assessed at a central laboratory using the VENTANA PD-L1 (SP263) assay and the tumor area positivity (TAP) scoring algorithm. Randomization was stratified according to a TAP score of ≥5% versus <5%, and overall survival was evaluated as a primary endpoint in both the TAP ≥5% population and all randomized patients. At the final analysis, median overall survival was 16.4 versus 12.8 months in the TAP ≥5% population (HR 0.71; 95% CI, 0.58–0.86) and 15.0 versus 12.9 months in the intention-to-treat population (HR 0.80; 95% CI, 0.70–0.92) (28). CPS scoring was subsequently performed retrospectively for exploratory analyses; in the CPS ≥1 subgroup, median overall survival was 15.1 versus 12.9 months (HR 0.78; 95% CI, 0.67–0.91) (29). Therefore, CPS should not be regarded as the prospectively specified patient-selection or stratification criterion of RATIONALE-305. Tislelizumab was approved by the FDA in December 2024 for the first-line treatment of unresectable or metastatic HER2-negative gastric/GEJ adenocarcinoma with PD-L1 expression ≥1 and is listed by the NCCN as a preferred first-line option for PD-L1 CPS ≥1, with a category 1 recommendation for CPS ≥5 (30). These findings suggest that PD-L1 expression, when interpreted using the relevant assay, scoring algorithm, and treatment-specific cutoff, may help estimate systemic-treatment sensitivity and identify a potential window for surgical reassessment. However, neither CPS nor TAP has been validated as an independent criterion for conversion surgery.

Nevertheless, PD-L1 expression, regardless of the scoring system used, cannot independently determine surgical indications. Its clinical value in conversion therapy mainly lies in predicting response depth and helping identify a potential surgical window. For patients with higher PD-L1 expression according to the relevant treatment-specific scoring algorithm and cutoff, R0 resection may be reconsidered through multidisciplinary assessment when marked regression of primary and metastatic lesions is achieved, peritoneal disease is stable or converts to negative, no new metastases appear, and the patient’s general condition remains adequate. In contrast, the potential for successful conversion in patients with low or negative PD-L1 expression, as determined using the relevant treatment-specific scoring algorithm and cutoff, may depend more strongly on chemosensitivity, tumor burden, and metastatic pattern.

#### 3.2.3 Stratification value of MSI/MMR in conversion therapy for gastric cancer

MSI-H/dMMR is one of the gastric cancer subtypes most strongly associated with immunotherapy sensitivity. Defective DNA mismatch repair leads to microsatellite instability, high tumor mutational burden, increased neoantigen production, and enhanced tumor immunogenicity (31). In clinical practice, dMMR is identified by immunohistochemical loss of MLH1, PMS2, MSH2, or MSH6, whereas MSI status can be assessed using polymerase chain reaction or next-generation sequencing. According to the TCGA, MSI gastric cancer is defined as a discrete subtype associated with genomic hypermutation, an immune-enriched tumor microenvironment, and activation of immune checkpoint signaling (32). An individual patient data meta-analysis further established MSI-H as a favorable prognostic biomarker in resectable gastric cancer and suggested limited benefit from perioperative chemotherapy in this subgroup (33).

Based on evidence from advanced and perioperative studies rather than conversion-specific randomized trials, we interpret MSI-H/dMMR as a marker of strong immunotherapy sensitivity that may facilitate subsequent surgical reassessment. In the NEONIPIGA study, all 29 patients who underwent surgery achieved R0 resection, and 17 of 29 patients (58.6%) achieved a pathological complete response after neoadjuvant nivolumab plus ipilimumab (34). These findings provide clinical evidence of marked immunotherapy sensitivity in MSI-H/dMMR gastric cancer. However, because NEONIPIGA predominantly enrolled patients with resectable disease, its results support the biological plausibility of conversion rather than directly demonstrating the efficacy of conversion surgery in metastatic gastric cancer. Emerging evidence also supports the investigation of non-operative management after an immunotherapy-induced clinical complete response (cCR) in selected patients with MSI-H/dMMR gastric or gastroesophageal junction adenocarcinoma. In cohort 2 of the INFINITY trial, 13 of 17 assessable patients achieved a cCR and entered non-operative management; one patient subsequently developed local regrowth and underwent salvage surgery, and the 12-month gastrectomy-free survival rate was 64.2% (35). Reflecting these findings, the current NCCN Clinical Practice Guidelines in Oncology for Gastric Cancer (Version 3.2026) recognize non-operative management as an option that may be considered for carefully selected patients with MSI-H/dMMR disease who achieve a rigorously confirmed cCR after immune-checkpoint blockade (30). Such an approach should be discussed by an experienced multidisciplinary team and requires intensive multimodal surveillance. In the INFINITY study, response assessment included cross-sectional imaging, endoscopic ultrasonography with pathological reassessment, and liquid-biopsy evaluation, with reassessment every 12 weeks for 2 years (35). Separately, a 2025 pooled individual-patient analysis reported a substantially higher pathological complete response rate with neoadjuvant immune-checkpoint inhibitors than with FLOT among resected patients with dMMR/MSI-H gastroesophageal adenocarcinoma (61.9% vs. 3.7%) (36). Nevertheless, these findings are derived primarily from small, non-randomized cohorts of initially resectable disease and do not establish routine omission of gastrectomy or direct applicability to initially unresectable conversion-therapy populations. Surgery or salvage surgery remains appropriate when cCR cannot be securely established, residual disease is suspected, or local regrowth occurs.

However, MSI/MMR status alone does not define surgical indications. Its value lies mainly in predicting immunotherapy response and identifying patients with potential for conversion surgery. For initially unresectable, borderline resectable, or oligometastatic MSI-H/dMMR disease, surgery may be considered when primary and metastatic lesions remain controlled, peritoneal status is clearly assessed, no new uncontrollable metastases appear, and R0 resection is considered feasible. Because radiological residual lesions after immunotherapy may not reflect true pathological activity, surgical timing should be determined dynamically by integrating imaging, endoscopic biopsy, tumor markers, laparoscopic exploration, and peritoneal lavage cytology.

#### 3.2.4 Stratification value of CLDN18.2 in conversion therapy for gastric cancer

CLDN18.2 is an emerging therapeutic target in gastric cancer and is particularly relevant for HER2-negative disease. As a gastric-specific isoform of the tight junction protein claudin-18, CLDN18.2 is normally located in gastric mucosal epithelial tight junctions. During tumorigenesis, impaired epithelial polarity may increase the surface accessibility of CLDN18.2, supporting its use as a target for antibody-directed therapy (37). Similar to HER2, CLDN18.2 expression may be heterogeneous and discordant between primary and metastatic lesions; therefore, standardized IHC testing, with multisite or metastatic lesion sampling when necessary, is important for accurate stratification. In the SPOTLIGHT (9) and GLOW (38) trials, CLDN18.2 positivity was determined by immunohistochemistry and defined as moderate-to-strong membranous staining in at least 75% of viable tumor cells.

Earlier phase II evidence from the FAST trial showed that adding zolbetuximab to EOX improved progression-free and overall survival in patients with CLDN18.2-positive advanced disease (39). SPOTLIGHT provided phase III evidence that zolbetuximab plus mFOLFOX6 improves survival outcomes in the first-line treatment of CLDN18.2-positive, HER2-negative unresectable or metastatic gastric or gastroesophageal junction adenocarcinoma (9). The GLOW trial further confirmed the survival benefit of zolbetuximab combined with CAPOX, supporting CLDN18.2 as an important first-line stratification biomarker in HER2-negative advanced gastric cancer (38). In addition, CLDN18.2-targeted ADCs, such as IBI343 and CMG901, have shown preliminary antitumor activity and manageable safety, but their role in initial conversion therapy remains investigational (40, 41). Beyond chemotherapy doublets, ILUSTRO cohort 4 reported a median PFS of 14.8 months and an ORR of 62.1% with first-line zolbetuximab plus mFOLFOX6 and nivolumab in unresectable CLDN18.2-positive disease; the randomized phase III LUCERNA trial (NCT06901531) is evaluating zolbetuximab plus pembrolizumab and chemotherapy (42). However, these data are not conversion-therapy-specific and do not yet define surgical indications.

**Figure 2:**
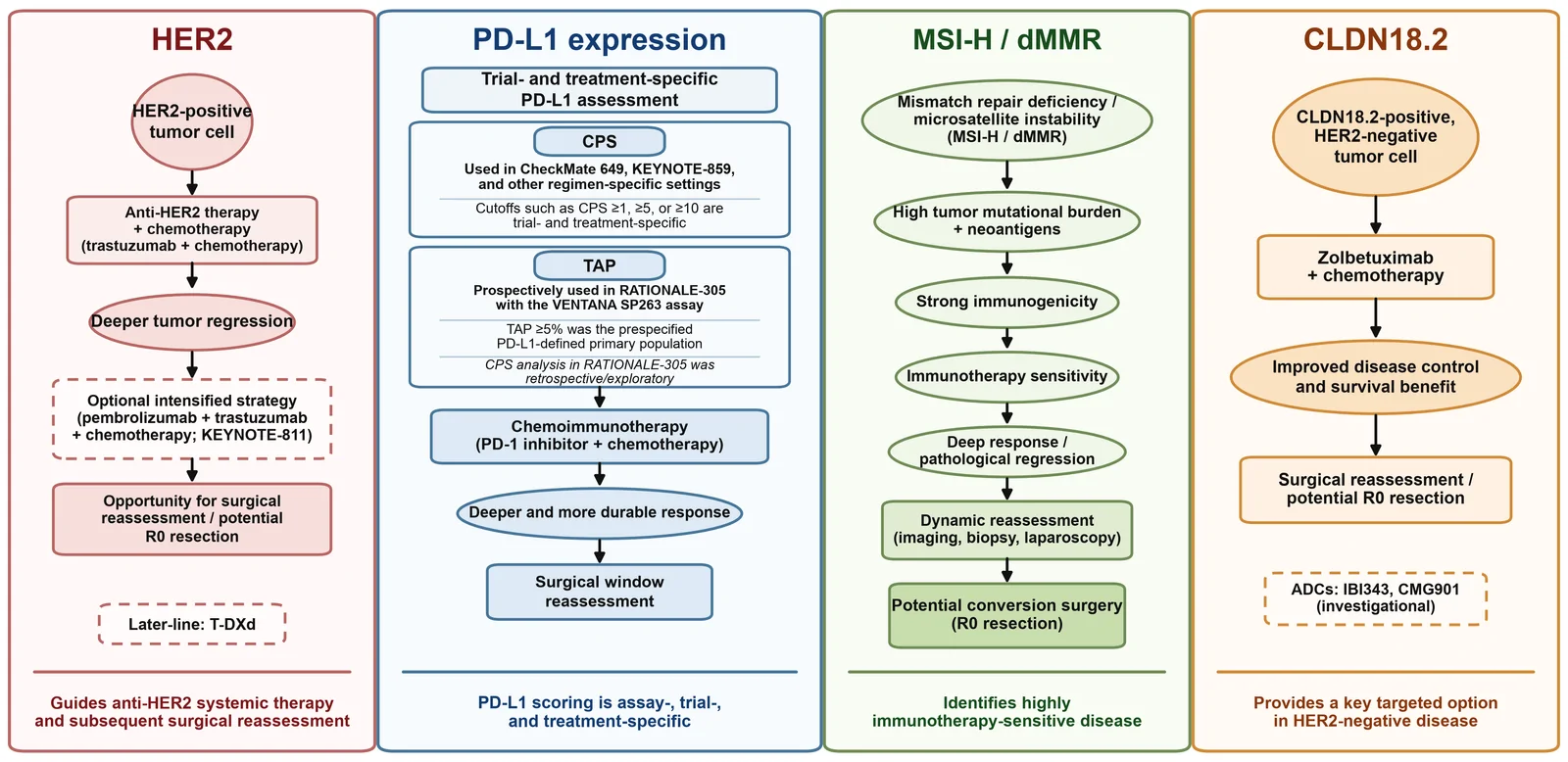
Biomarker-guided treatment selection and conversion potential in advanced gastric cancer. The figure illustrates how HER2, PD-L1 expression assessed using trial- and treatment-specific scoring algorithms, including CPS and TAP, MSI-H/dMMR, and CLDN18.2 may guide systemic treatment and subsequent surgical reassessment. CPS, combined positive score; TAP, tumor area positivity; dMMR, deficient mismatch repair; MSI-H, microsatellite instability-high; R0, microscopically margin-negative resection. CPS and TAP are distinct PD-L1 scoring algorithms and their cutoffs should not be considered interchangeable. The links from systemic response to surgical reassessment and potential R0 resection are conceptual because the pivotal biomarker trials did not directly evaluate conversion-specific endpoints.

Based on advanced-disease trials rather than conversion-specific studies, we interpret CLDN18.2 positivity as a potential basis for improving systemic disease control and enabling surgical reassessment in selected HER2-negative patients. In SPOTLIGHT, zolbetuximab plus mFOLFOX6 prolonged median progression-free survival from 8.67 to 10.61 months compared with placebo plus mFOLFOX6 (HR 0.75) (9), while GLOW provided additional evidence supporting zolbetuximab combined with chemotherapy (38). For patients with CLDN18.2-positive, HER2-negative, locally advanced unresectable or oligometastatic disease, improved systemic control may create an opportunity for reassessment of resectability. However, CLDN18.2 status alone cannot determine surgical eligibility, and conversion surgery should still require control of both primary and metastatic lesions, absence of new disease, and multidisciplinary confirmation that R0 resection is feasible (**Figure 2**).

## 4 Integrated application of four molecular biomarkers in clinical stratification and decision-making pathways

In gastric cancer conversion therapy, HER2, PD-L1, MSI/MMR, and CLDN18.2 may inform a provisional framework for pretreatment stratification, systemic-treatment selection, and surgical reassessment, although their application is based predominantly on evidence extrapolated from advanced-disease settings (43). MSI-H/dMMR tumors may favor immunotherapy-based strategies; HER2-positive disease supports anti-HER2 therapy plus chemotherapy, with possible addition of immunotherapy when PD-L1 is positive; and CLDN18.2-positive, HER2-negative disease may benefit from CLDN18.2-targeted therapy plus chemotherapy. In biomarker-negative or low-expression tumors, conversion potential depends more on chemosensitivity, tumor burden, and metastatic extent (32, 44). However, biomarkers cannot replace surgical judgment. Conversion surgery should be considered only after effective systemic control, absence of new uncontrolled metastases, stable or negative peritoneal disease, and confirmation that R0 resection is technically feasible (2, 8). Biomarker results should be interpreted jointly rather than sequentially because actionable biomarkers are not mutually exclusive. The 2026 ASCO Guideline Update recommends that HER2, PD-L1, MSI/MMR, and CLDN18.2 results should be available before treatment planning whenever feasible (45). In a recent US real-world cohort, 13.4% of patients evaluated for multiple biomarkers had at least two actionable findings. Treatment concordance was higher for HER2-positive disease (62%) than for PD-L1- or CLDN18.2-positive disease (<37%), highlighting the uncertainty created by biomarker overlap (46). A pragmatic prioritization framework may therefore be considered. MSI-H/dMMR status should strongly favor the inclusion of immunotherapy. In pMMR/MSS disease, HER2-positive tumors should remain on a HER2-directed pathway, with pembrolizumab added when PD-L1 CPS is ≥1; coexisting CLDN18.2 positivity does not currently displace HER2-directed treatment because phase III evidence for zolbetuximab is limited to HER2-negative disease. For HER2-negative tumors, PD-L1-positive/CLDN18.2-negative disease favors chemoimmunotherapy, whereas CLDN18.2-positive tumors with PD-L1 CPS <1 favor zolbetuximab plus chemotherapy. When PD-L1 and CLDN18.2 are both positive, no head-to-head evidence establishes a preferred regimen. A higher CPS may strengthen the rationale for chemoimmunotherapy, whereas a lower CPS may favor zolbetuximab; toxicity profiles, symptom burden, comorbidities, patient preference, and treatment access should also be considered (45). Simultaneous PD-1 and CLDN18.2 targeting remains investigational (42). In conversion therapy, this framework remains an extrapolation from advanced-disease guidance rather than a validated surgical algorithm. Regimen selection should aim for deep and durable control of all disease sites, followed by multidisciplinary reassessment of R0 resectability.

## 5 Research gaps and future directions

Although HER2, PD-L1, MSI/MMR, and CLDN18.2 may support a provisional molecular framework for gastric cancer conversion therapy, current evidence remains limited (30). Although conversion-specific prospective data are emerging, current evidence remains dominated by small, single-arm phase II studies and extrapolation from palliative trials; controlled studies using R0 resection, pathological response, and postoperative survival as prespecified endpoints are still needed. Biomarker heterogeneity also limits the reliability of single-sample testing. Emerging targets and strategies, including FGFR2b, TROP2, TMB, EBV status, antibody-drug conjugates, bispecific antibodies, and cellular therapies, may expand future treatment options (47, 48). However, their clinical value requires further validation. Therefore, the four core biomarkers remain the main basis for current stratified treatment and surgical reassessment (45, 49).

## 6 Conclusion

HER2, PD-L1, MSI/MMR, and CLDN18.2 may provide a preliminary framework for treatment stratification and surgical reassessment in gastric cancer conversion therapy. However, this framework is derived primarily from evidence generated in palliative and neoadjuvant settings rather than from conversion-therapy-specific trials. In selected patients with initially unresectable, borderline resectable, or oligometastatic disease, biomarker status may help guide induction therapy and subsequent surgical reassessment. HER2 positivity may support anti-HER2 combination therapy, PD-L1 expression, as assessed using trial- and treatment-specific scoring algorithms such as CPS or TAP, may help identify patients who benefit from immunotherapy combined with chemotherapy, MSI-H/dMMR suggests greater sensitivity to immunotherapy, and CLDN18.2 offers a targeted option for HER2-negative disease.

However, molecular biomarkers should not replace surgical evaluation. The goal of conversion therapy is not only tumor shrinkage on imaging, but also durable disease control, complete tumor removal, and potential long-term survival benefit. Therefore, clinical decisions should combine pretreatment biomarker testing, repeated response assessment, and multidisciplinary surgical review. During treatment, imaging, endoscopy, laparoscopy, peritoneal lavage cytology, tumor markers, and repeat biopsy when needed may help evaluate response, emerging resistance, and peritoneal disease status.

After systemic treatment, surgery should be considered only when the primary tumor and metastatic lesions are well controlled, no new uncontrolled metastases have appeared, and R0 resection is technically feasible. In the future, circulating tumor DNA, imaging-based analysis, artificial intelligence, and new targeted agents may further improve individualized conversion strategies (50, 51). Prospective conversion-therapy-specific trials incorporating conversion-surgery rate, R0 resection, pathological response, disease-free survival, and overall survival as predefined endpoints are required to validate the clinical utility of this proposed framework.
